# Persistence of microbial communities including *Pseudomonas aeruginosa* in a hospital environment: a potential health hazard

**DOI:** 10.1186/1471-2180-14-118

**Published:** 2014-05-08

**Authors:** Pedro Miguel de Abreu, Pedro Geadas Farias, Gabriel Silva Paiva, Ana Maria Almeida, Paula Vasconcelos Morais

**Affiliations:** 1 Instituto Piaget, Enxerim 8300-025, Silves, Portugal; 2 IMAR – Marine and Environmental Research Centre, University of Coimbra, 3004-517 Coimbra, Portugal; 3 Department of Life Sciences, FCTUC, University of Coimbra, 3004-517 Coimbra, Portugal

**Keywords:** Pseudomonas aeruginosa, Hospital environment, Surface microbial colonization, Stenotrophomonas maltophilia

## Abstract

**Background:**

The persistence of microbial communities and how they change in indoor environments is of immense interest to public health. Moreover, hospital acquired infections are significant contributors to morbidity and mortality. Evidence suggests that, in hospital environments agent transfer between surfaces causes healthcare associated infections in humans, and that surfaces are an important transmission route and may act as a reservoir for some of the pathogens.

This study aimed to evaluate the diversity of microorganisms that persist on noncritical equipment and surfaces in a main hospital in Portugal, and are able to grow in selective media for *Pseudomonas,* and relate them with the presence of *Pseudomonas aeruginosa*.

**Results:**

During 2 years, a total of 290 environmental samples were analyzed, in 3 different wards. The percentage of equipment in each ward that showed low contamination level varied between 22% and 38%, and more than 50% of the equipment sampled was highly contaminated. *P. aeruginosa* was repeatedly isolated from sinks (10 times), from the taps’ biofilm (16 times), and from the showers and bedside tables (two times). Two ERIC clones were isolated more than once. The contamination level of the different taps analyzed showed correlation with the contamination level of the hand gels support, soaps and sinks. Ten different bacteria genera were frequently isolated in the selective media for *Pseudomonas*. Organisms usually associated with nosocomial infections as *Stenotrophomonas maltophilia*, *Enterococcus feacalis*, *Serratia nematodiphila* were also repeatedly isolated on the same equipment.

**Conclusions:**

The environment may act as a reservoir for at least some of the pathogens implicated in nosocomial infections. The bacterial contamination level was related to the presence of humidity on the surfaces, and tap water (biofilm) was a point of dispersion of bacterial species, including potentially pathogenic organisms*.* The materials of the equipment sampled could not be related to the microbial contamination level. The presence of a disinfectant in the isolation medium suggests that the number of microorganism in the environment could be higher and shows the diversity of disinfectant resistant species. The statistical analysis suggests that the presence of bacteria could increase the risk of transmission by hand manipulation.

## Background

Hospitals are environments where both, infected and non-infected people, group. How microbial communities persist and change in indoor environments is of immense interest to public health. Recent work showed that humans alter the microbiome in a space when they occupy that space [1]. Building materials and equipment seem also to influence the community composition. For instance, recent studies show that materials made of copper have lower surface burden than stainless steel or plastic materials [2,3].

The potential for contracting a microbial pathogen is highest within a hospital environment [4]. Hospital acquired infections (HAI) are significant contributors to morbidity and mortality, with no values attributed (in http://www.who.int/en/), the Center for Disease Control defined the baselines for HAI as those occurring more than 48 h/72 h after healthcare admission and within 10 days after hospital discharge [5]. Despite the lack of direct evidence to prove that environmental contaminants are responsible for HAIs, there is an increasing evidence suggesting that the environment may act as a reservoir for at least some of the pathogens causing HAIs [6-9]. Therefore, by touching contaminated surfaces and noncritical equipment, hands may acquire and transfer microorganisms to other inanimate objects or to patients [10,11]. Guidelines on treatment of surfaces in hospitals take into account parameters which are considered to be relevant for preventing the transmission of nosocomial pathogens, such as the type of ward or the expected frequency of hand contact with a surface [12].

The presence of susceptible patients in hospital makes more important the adverse impact of the environment on the incidence of health-care–associated infections. Data from the World Health Organization show that nowadays in every 100 hospitalized patients 7 to 10 are expected to contract, at least, one health care-associated infection [13]. Hospital-associated pathogens are commonly found on physician’s and nursing staff’s clothing [14,15], cell phones [16,17], stethoscopes [18], computer keyboards [19], telemetry leads [20], electronic thermometers [21], blood-pressure cuffs [22], and gels for ultrasound probes [23]. The outbreaks of *Pseudomonas aeruginosa*[24] linked to water and aqueous solutions used in health-care facilities are examples of these health-care–associated infections. Additionally, clinically important opportunistic organisms linked to water are *Pseudomonas* spp., *Acinetobacter baumannii Burkholderia cepacia*, *Ralstonia pickettii*, *Stenotrophomonas maltophilia*, and *Sphingomonas* spp. Modes of transmission for waterborne infections include direct contact, ingestion of water, indirect-contact, inhalation of aerosols dispersed from water sources and aspiration of contaminated water [12].

In this work, we hypothesizes that the existing microbial communities, associated with the surfaces and noncritical equipment, do influence the colonization of other organisms as *Pseudomonas aeruginosa*, one of the major agents for nosocomial infections, and make them available to be transferred. The aim of the present work was to evaluate the diversity of microorganisms able to grow in selective medium for *Pseudomonas* including *P. aeruginosa* that persists on noncritical equipment and surfaces in a hospital.

## Results

### General level of contamination of the equipment in each ward

**Figure 1:**
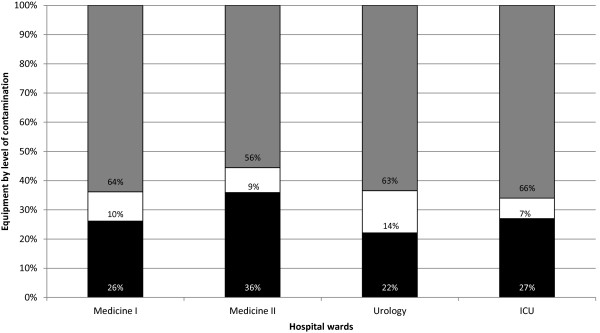
**Percentage of equipment with different levels of contamination.** Low level contamination (blue), medium level of contamination (red) and high level of contamination (green).

The study included 4 of wards, sampled during 9 months, between February 2010 and September 2011. The samples were recovered from 10 cm^2^ area using a swab soaked in Tryptic Soy Broth. A total of 290 environmental samples were analyzed for bacterial colonization. The samples were plated in Pseudomonas isolation agar medium (PIA) which is a selective medium used for the isolation of *P. aeruginosa* and other *Pseudomonas* species [25]. The number of colonies growing on PIA medium varied in the different equipment sampled. However, a pattern could be defined when considering three classes of level of contamination defined from the amount of counts obtained on PIA medium, based on the accuracy of plate counts enumeration [26]. The first level of contamination included equipment with less than 10 CFU per plate (low contaminated), 10 CFU per plate are considered the minimum CFUs for statistical significance, the second included equipment with CFU between 10 and 200 CFU per plate (medium contaminated), and the equipment with more than 200 CFU per plate were included in the third level (high contaminated), CFU counts over 200 are considered uncountable due to spatial growth restrictions.The percentage of equipment in each ward that showed low contamination level varied between 22% and 38% (Figure 1). Equipment with a surface number of CFU varying between 10 and 200 CFU were a minority in all wards (maximum 15%) and, in all wards, more than 50% of the equipment sampled had more than 200 CFU per sample. The level of colonization of the equipment was similar in the UCI compared to the Medicine I and II and Urology wards.

The majority of the samples collected in taps and sinks showed high level of contamination (Table 1). This pattern of contamination was observed during the 2 years of sampling. High level of contamination was also detected in the showers but in a low number of samples. On the other hand, contamination on surface countertops and trays was detected only in spring samples (March 2010 and April 2011). The noncritical equipment manipulated mostly by the medical personnel as workbenches, stethoscopes and other medical equipment was either not contaminated or low contaminated (six samples in 2 years), but when the oxygen flask was found contaminated (one sample), the contamination level was high.

**Table 1:** Percentage of microbial colonization obtained in the different equipment during the two years sampling

**Colony Count**	**Feb 2010**		**Apr 2010**		**Jun 2010**		**Oct 2010**		**Dec 2010**		**Feb 2011**		**Mar 2011**		**Jun 2011**		**Sep 2011**	
	**Low**	**High**	**Low**	**High**	**Low**	**High**	**Low**	**High**	**Low**	**High**	**Low**	**High**	**Low**	**High**	**Low**	**High**	**Low**	**High**
Sink (porcelain)	15.4	47.6	15.8	46.2	27.3	34.3	-	34.2	23.1	30.0	25.0	54.2	12.5	58.8	12.5	62.2	28.6	52.9
Tap	7.7	4.8	-	23.1	36.4	34.3	25.0	31.6	38.5	45.0	31.3	37.5	50.0	26.5	5-	29.7	42.9	29.4
Countertop (sinks)	-	-	15.8	15.4	-	-	-	2.6	-	-	-	-	12.5	2.9	-	-	-	-
Workbench	15.4	4.8	15.8	7.7	-	-	-	10.5	7.7	-	-	4.2	12.5	-	12.5	-	7.1	-
Shower (+handrail)	7.7	14.3	-	-	-	8.6	-	13.2	-	5.0	6.3	4.2	-	5.9	-	5.4	-	2.9
Bedside table	15.4	4.8	10.5	7.7	27.3	5.7	12.5	2.6	7.7	-	12.5	-	-	2.9	-	-	14.3	-
Handrail bed (+bed)	-	4.8	5.3	-	-	-	-	2.6	-	-	-	-	-	-	-	-	-	-
Serum support	-	-	10.5	-	-	-	-	-	-	-	-	-	-	-	-	-	-	2.9
Oxygen flask	-	4.8	-	-	-	-	-	-	7.7	-	-	-	-	-	-	-	-	-
Stethoscope	7.7	-	-	-	-	-	12.5	-	-	-	-	-	-	-	-	-	-	-
Equip bedside	-	-	-	-	-	-	-	-	-	-	-	-	-	-	-	-	-	-
Medical equipments	7.7	9.5	15.8	-	-	-	12.5	-	7.7	-	-	-	-	-	-	-	-	2.9
Tray	23.1	4.8	5.3	-	9.1	5.7	12.5	-	7.7	5.0	12.5	-	-	-	-	2.7	-	5.9
Hand gel/soap	-	-	-	-	-	11.4	25.0	2.6	-	15.0	12.5	-	-	-	25.0	-	7.1	-
Table (meal/work)	-	-	5.3	-	-	-	-	-	-	-	-	-	12.5	2.9	-	-	-	2.9

Results show only high and low levels of contamination per sampling.

**Figure 2:**
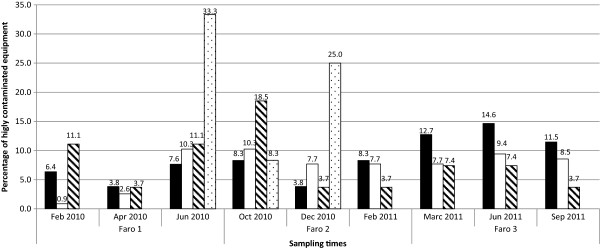
**Variation of the number of highly contaminated equipment; porcelain sink (****), tap (****), shower and handrail (****), hand gel/soap (****); during the sampling period per group of equipment selected based on the persistence and level of contamination.**

The contamination level of the different taps analyzed showed a correlation of 0.9 and 0.8 with the contamination level of the hand gels support and with the soaps and sinks, respectively (p < 0.05). The correlation of tap contamination was only of 0.6 with the samples collected in the showers (p < 0.05). On the other hand, tap contamination level correlated in less than 0.2 (p < 0.01) with the contamination of the workbenches and the trays of the clinical personnel, and with the contamination of the bed and bedside table.The equipment that showed persistently a high level of contamination were the surface of sinks, the taps, the hand gels and soaps and the showers. The number of highly contaminated samples from these equipment increased in samples collected during summer and fall, in both years, except for the samples collected on hand gels. The number of positive samples on hand gel/soap was high but only during a short period (until the end of 2010) (Figure 2).

### Diversity of isolates recovered on the equipment and identified by 16S rRNA gene sequence

PIA medium recovered strains of *P. aeruginosa* but also strains belonging to 10 different bacterial genera, although its formulation was conceived to be a selective medium for *Pseudomonas*. The medium was able to isolate bacteria belonging to the family *Pseudomonas* as well as gram positive bacteria as *Bacillus aryabhattai* and *Neisseria subflava*.

Strains of *P. aeruginosa* were isolated in all equipment showing a high number of samples with high level of contamination (Table 2). *P. aeruginosa* was repeatedly isolated in the sinks (10 times), in the biofilm of the taps (16 times), in the showers and bedside tables (two times). The analysis of REP profiles suggest the existence of 2 clones. Clone A included 2 strains from sampling time F4 (F4-42 and F4-44), isolated from a sink and a tap, and from sampling time F3 (F3-6) also from a tap but from a different ward. Clone B included two strains (F4-6b and F7-6a) from different sampling times (F4 and F7) isolated from the same tap (Additional file 1: Figure S1).

**Table 2:** Diversity of bacteria isolated and identified by 16S rRNA gene sequencing

	**Samples showing fluorescence by month and year**									**Organisms isolated (number of strains)**
Month/Year	F 10	A 10	J 10	O 10	D 10	F 11	M 11	J 11	S 11	
Sink	3	6	4	4	7	16	8	9	10	*Acinetobacter pittii*
										*Bacillus aryabhattai*
										*Citrobacter braakii*
										*Citrobacter freundii*
										*Enterococcus faecalis*
										***Pseudomonas aeruginosa (10)***
										Pseudomonas beteli*
										*Pseudomonas hibiscicola*
										*Pseudomonas monteilii*
										*Pseudomonas mosselii*
										*Pseudomonas plecoglossicida*
										*Pseudomonas putida*
										*Pseudomonas taiwanensis*
										*Serratia nematodiphila*
										***Sphingobium yanoikuyae (2)***
										***Stenotrophomonas maltophilia (3)***
										*Stenotrophomonas rhizophila*
Tap	-	3	3	5	9	5	8	7	7	*Citrobacter braakii*
										***Enterococcus faecalis (2)***
										*Erwinia aphidicola*
										*Neisseria subflava*
										***Pseudomonas aeruginosa (16)***
										*Pseudomonas hibiscicola*
										*Pseudomonas monteilii*
										***Serratia nematodiphila (2)***
										***Stenotrophomonas maltophilia (6)***
Shower (Handrail)	1	1	2	1	1	1	-	2	-	***Pseudomonas aeruginosa (2)***
										*Pseudomonas plecoglossicida*
										*Pseudomonas monteilii*
Hand Gel (soap)	-	-	1	-	3	-	-	-	-	***Pseudomonas aeruginosa***
										Pseudomonas beteli*
										*Shewanella oneidensis*
										*Citrobacter freundii*
Workbench/ S. countertop	1	1	-	4	-	-	1	-	-	***Pseudomonas aeruginosa***
										Pseudomonas beteli*
Tray	-	-	2	-	2	-	-	-	2	***Pseudomonas aeruginosa***
Bedside Table	1	-	2	-	1	-	-	-	-	***Pseudomonas aeruginosa (2)***
										Pseudomonas beteli*
										*Pseudomonas monteilii*
Bedside equipment	-	-	-	-	-	-	-	1	-	***Pseudomonas aeruginosa***
Table (work/meal)	-	1	-	-	-	-	1	-	1	*Pseudomonas alcaligenes*
										*Pseudomonas putida*

(*- bacterial species isolated in different equipment).

The isolation of strains from the species *P. aeruginosa* was expected since the isolation conditions favoured its recovery. However, *Stenotrophomonas maltophilia*, *Enterococcus feacalis, Sphingobium yanoikuyae* and *Serratia nematodiphila* were also repeatedly isolated on the same equipment, on different times. Seven different species of *Pseudomonas* were isolated on the sinks surfaces. Some of these species were also isolated on other surfaces as *P. beteli* on hand gel/soap, workbench and bedside table. *P. montelli* was also isolated on the sink surfaces, taps, showers and bedside tables.

**Figure 3:**
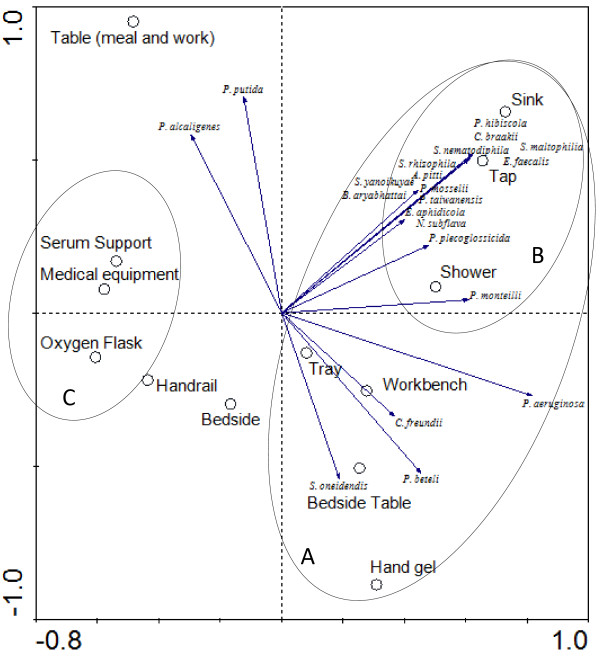
**PCA based on the level of contamination of the equipment and the bacterial diversity present, during the sampling period.** Samples grouped in moistened **(A)**, a sub-group of samples contacting with tap water **(B)** and in those manipulated mostly by the hospital personnel **(C)**; table for meal and work, handrail and bedside (equipment) were not grouped.

Some of the organisms isolated were already reported as pathogenic. This is the case of *Citrobacter braakii*, *C. freundii*, *E. faecalis*, *P. mosselii*, *P. putida*, *S. maltophilia*, *Neisseria subflava*, *P. alcaligenes* or isolated from hospital environment as *P. monteilii*.The principal component analysis was carried to correlate the level of contamination of the equipment with the bacterial diversity present during the sampling period. The cumulative percentage variance of species was 50.2. The PCA analysis grouped the samples in two major groups: moistened samples (A), with a sub-group of samples directly contacting with tap water (B) and samples manipulated mostly by the hospital personnel (C) (Figure 3); table for meal and work, handrail and bedside (equipment) were not grouped.

## Discussion

Microorganisms are ubiquitous in our environment, including indoor air, and do not necessarily constitute a health hazard. Depending on the individual, the concentration at which contamination becomes a threat to health is unknown [9]. Inanimate surfaces and noncritical equipment have often been described as the source for outbreaks of nosocomial infections [27-29]. The aim of this work was to evaluate, in a Portuguese hospital facility, the number and diversity of microorganisms that persist on inanimate surfaces and noncritical equipment, able to grow on the selective media for *P. aeruginosa* and relate them with the presence of the opportunistic pathogen *P. aeruginosa*.

Data is available on the microbial composition of dust from different environments, showing Gram-positive as dominants, with the most abundant phylum being Firmicutes [7]. However, other studies on the microbial diversity of the environmental surfaces are mainly evaluating the bacterial counts on cloths and other equipment from medical personnel [15].

In the present study, PIA medium was used to recover microorganisms from noncritical equipment and from surfaces, dry or wet. PIA is an isolation medium selective and differential for *P. aeruginosa,* since this species has innate resistance to low Irgasan concentrations [30]. Nevertheless, 10 different bacterial genera of Gram negative and Gram positive bacteria were isolated in the medium which seems to indicate that these organisms are resistant to the biocide and could possibly have multidrug efflux systems to extrude the antimicrobial Triclosan (Irgasan) as it occurs in *P. aeruginosa*[31]. This conclusion is supported by the detection of clonal isolates from different sampling times. The presence of this toxic in many household antibacterial products and antiseptics can probably select for microorganisms able to resist to low concentrations of this biocide [30]. Many Gram-negative species were isolated, which is according to previous reports showing that strains from *Acinetobacter* spp., *Klebsiella* spp., *Shigella* spp., *E. coli*, *P. aeruginosa*, or *S. marcescens* are able to survive for months on surfaces [32]. These species are among the most frequent isolates from patients with nosocomially acquired infections [32]. Moreover, all isolates from this work are resistant to the disinfectant Triclosan, on the other hand, not all the microorganisms present in the environment were isolated.

*P. aeruginosa* is described to persist from 6 hours to 16 months on surfaces and its persistence was related with humidity conditions [32,33]. *P. aeruginosa* was also found in the present work, as part of the microbial community of surfaces with high moister and also in the biofilm of taps. Even though, ubiquitous in the environment, the prevalence of this species in the community is less than in the hospital, and cases of severe community-acquired infection are rare [34]. *Pseudomonas* have been implicated in different clinical syndromes and diseases transmitted mostly directly by aerosols or indirectly by moist environmental surfaces via hands of health-care workers [12,35]. In the present work, biofilm tap water was the major environmental source of pseudomonads in the healthcare facility. This conclusion is in agreement with previous findings where biofilms, sink and patient room design were involved in the propagation of a *P. aeruginosa* outbreak [35]. Moreover, humidity (wet materials) improved the presence of high numbers of different bacteria species which are clinically important opportunistic organisms as other *Pseudomonas* as *P. mosselii*, *P. putida, P. alcaligenes, Citrobacter braakii*, *C. freundii*, *E. faecalis*, *S. maltophilia*, *N. subflava,* as found before [36,37].

In the hospital studied *S. maltophilia* was isolated nine times in the sinks and in the biofilm of the taps*, E. faecalis* and *S. nematodiphila* were repeatedly isolated, two times each, in tap water biofilms, and *S. marcescens* and *Enterobacter* spp. were also isolated during the present study. The described genera were reported to be responsible for healthcare–associated episodes of colonization, including respiratory and urinary tracks, bloodstream infections and pneumonia [5,12,38]. *E. faecalis*, *S. nematodiphila*, *S. marcescens* and *Enterobacter* spp. are commonly associated with transmission by hand carriage and hand transfer [39]

The different type of materials tested did not reveal a consistent (high or low) contamination level. Some investigators reported that the type of material has no influence on the persistence of bacteria, other described a longer bacterial persistence on plastic, others on steel, or a shorter survival on copper [2,3,32,40]. The statistical analysis of the results based on the contamination level, number of times contaminated and type of material, grouped samples on the base of the group of persons that manipulated the equipment, on the presence or absence of humidity and contact with tap water, but not based on their type of material. This study used a selective medium with a disinfectant to evaluate the microbial colonization of the surfaces and noncritical equipment, revealing the prevalence of a diverse group of microbial species with mechanisms of resistance to the antiseptic, most of them with potential to be involved in nosocomial infections.

## Conclusion

The potential for contracting a microbial pathogen is highest within a hospital environment and hospital acquired infections are significant contributors to morbidity and mortality. Despite the lack of direct evidence to prove that environmental contaminants are responsible for hospital acquired infections, there is an increasing evidence suggesting that the environment may act as a reservoir for at least some of the pathogens causing nosocomial infections. This work showed that many different bacterial species can persist on surfaces for months and years. The level of bacterial contamination was related with the presence of humidity on the surface, and tap water (biofilm) was a point of dispersion of bacterial species, usually involved in nosocomial infections as *Pseudomonas aeruginosa*, *Stenotrophomonas maltophilia* and *Enterococcus feacalis*. Their presence in the environment, as it seems to be pointed by the analysis of the diversity, increases the risk of transmission to the different materials during hand manipulation.

## Methods

### Sampling (ICU, Medicine I, Medicine II and Urology)

The study was carried out at the Hospital de Faro, Portugal, which serves a resident population of about 253 thousand people and this value may double or triple the population seasonally (in http://www.hdfaro.min-saude.pt/site/index.php). Between January 2010 and September, 2011, the hospital was evaluated 12 times (sampled each two months) and four different wards were investigated for environmental contamination of the following surfaces and equipment: sink, tap (biofilm), surface countertop and workbench of the nurses area, shower (and handrail), bedside table, handrail bed (including bed), serum support, oxygen flask, stethoscope, equipment at bedside, other medical equipment, tray used by nurses, hand gel/soap, table (meal and work). The equipment considered in this study is included in the category of noncritical hospital objects and surfaces. These items have been said to pose no risk to patients, nevertheless, these surfaces and equipment are frequently touched by hand can contribute to the spread of healthcare-associated pathogens as *Pseudomonas aeruginosa*, *Staphilococus aureus*, or *Acinetobacter baumanii*. The evaluation was performed in wards of the Medical Unit I and II, Urology and Intensive Care Unit.

Samples were collected in the wards, always in the same period of the day, at the end of the morning and during lunch time, after the medical visits and treatments, and also sometime after a ward cleaning. Swabs were used for collecting the organisms present in an area of 10X10 cm of each surface. Taps were sampled by removing the biofilm. The swabs were first humidified in Tryptic Soy Broth (TSB - Oxoid, Basingstoke, Hampshire, England), then used to sample, and transported in 2 ml of TSB tubes and then processed in the laboratory after 3 h shaking.

### Number of cultivable microorganisms on equipment and bacterial isolation

Each volume of transporting broth containing single swabs was vortexed for 1 min. A total of 290 environmental samples were analysed for bacterial colonization by inoculating 0.1 ml of the swab suspension in Pseudomonas Isolation Agar (PIA) (Difco). PIA is a selective medium including the antibiotic Irgasan for the isolation of *Pseudomonas* and differentiating *Pseudomonas aeruginosa* from other pseudomonads on the basis of pigment formation. Samples were incubated 24 h at 30°C, and evaluated after this period for total counts and for the presence of colonies with fluorescence under UV light. All colonies showing fluorescence were isolated and purified. From plates positive for fluorescence, a significant number of non-fluorescent colonies were also isolated.

### 16S rRNA gene sequence identification of the isolates

DNA from each isolate was obtained using the protocol from Pitcher *et al*. [41] with the following modifications: an extra washing step with a second volume of 24:1 (v/v) of chloroform/isoamyl-alcohol and an additional centrifugation step for 15 min at 13 200 rpm were added. Amplification of the nearly full-length 16S rRNA gene sequence from each DNA was performed by PCR with primers 27 F (5′-GAG TTT GAT CCT GGC TCA G – 3′) and 1525R (5′ – AGA AAG GAG GTG ATC CAG CC – 3′) [42]. The PCR reaction was performed according to Proença *et al.*[43]. Briefly, 30 μl reaction mix was amplified using PCR with 30 cycles: 1 min at 94°C, 1 min at 53°C, and 1 min at 72°C. The 1500-bp PCR products were purified using the JET Quick PCR Purification Spin Kit (Genomed GmbH, Löhne, Germany) according to the manufacturer’s instructions.

All sequences were compared with sequences available in the NCBI database using BLAST network services [44]. Sequences were initially aligned with the CLUSTAL X program [45], visually examined, and relocated to allow maximal alignment. The method of Jukes and Cantor [46] was used to calculate evolutionary distances. Phylogenetic dendrograms were than constructed by the neighbour-joining method using the MEGA4 package [47].

### REP typing of *P. aeruginosa* strains

A primary screen of all isolates was performed by Random Amplification of Polymorphic DNA (RAPD) using DNA amplification reactions in a total volume of 30 μl according to Santos *et al.* 2012 [48]. The RAPD patterns were visually analysed.

Clones of *P. aeruginosa* strains were identified by ERIC-PCR. Polymerase chain reaction, both reaction mix and amplification cycle, followed the protocol outlined by Syrmis, *et al*. 2004 [49]. Samples were loaded on a 1% agarose gel with TAE and runned at 75 V for 1 h, at room temperature.

### Statistical analysis

The correlation (Pearsons) between samples, based on the contamination level, was performed by using Microsoft Excel. Principal Component Analysis was used to analyse the relationships between the level of contamination of the equipment, the type of equipment and the sampling time using the software package CANOCO (version 4.5).

### Nucleotide sequence accession numbers

The 16S rRNA gene sequences of the isolates reported in this study (except strain Faro2_34) have been deposited in EMBL database under the accession numbers from KF792126 to KF792306.

## Competing interests

The authors declare that they have no competing interests.

## Authors’ contributions

PA performed the sample collection and laboratory work including DNA extraction, bacteria identification and antibiotic testing. PF performed the sequence submission to data bank and in the manuscript. GP performed sampling and bacterial isolation. AA contributes in the study design and manuscript revising. PVM conceived of the study, participated in the overall design and coordination and the manuscript. All authors read and approved the final manuscript.

## Supplementary Material

Additional file 1: Figure S1 ERIC-PCR profiling of: *Pseudomonas aeruginosa* strains f2-3b, faro2 29a, faro3 3a, faro3 6, faro3 10a, faro3 16a, faro4 6b, faro4 42, faro4 44, faro4 47a, faro6 39a, faro 7 6a and faro7 10, faro 7 17 and faro8 20, figure **a)** from left to right. On figure **b)** the strains *P. aeruginosa* faro8 26, faro8 36a, faro8 40a, faro6 5a, faro6 42, faro7 20c and faro8 6. Samples loaded on electrophoresis gel 1% agarose, 70 V, 60 min, stained with ethidium bromide. Click here for file
