# Supplementary figures and images for: Persistence of microbial communities including Pseudomonas aeruginosa in a hospital environment: a potential health hazard
Source: BMC Microbiol. 2014 May 8;14:118. doi: 10.1186/1471-2180-14-118 (PMC4049484; doi:10.1186/1471-2180-14-118)

## Slide 1
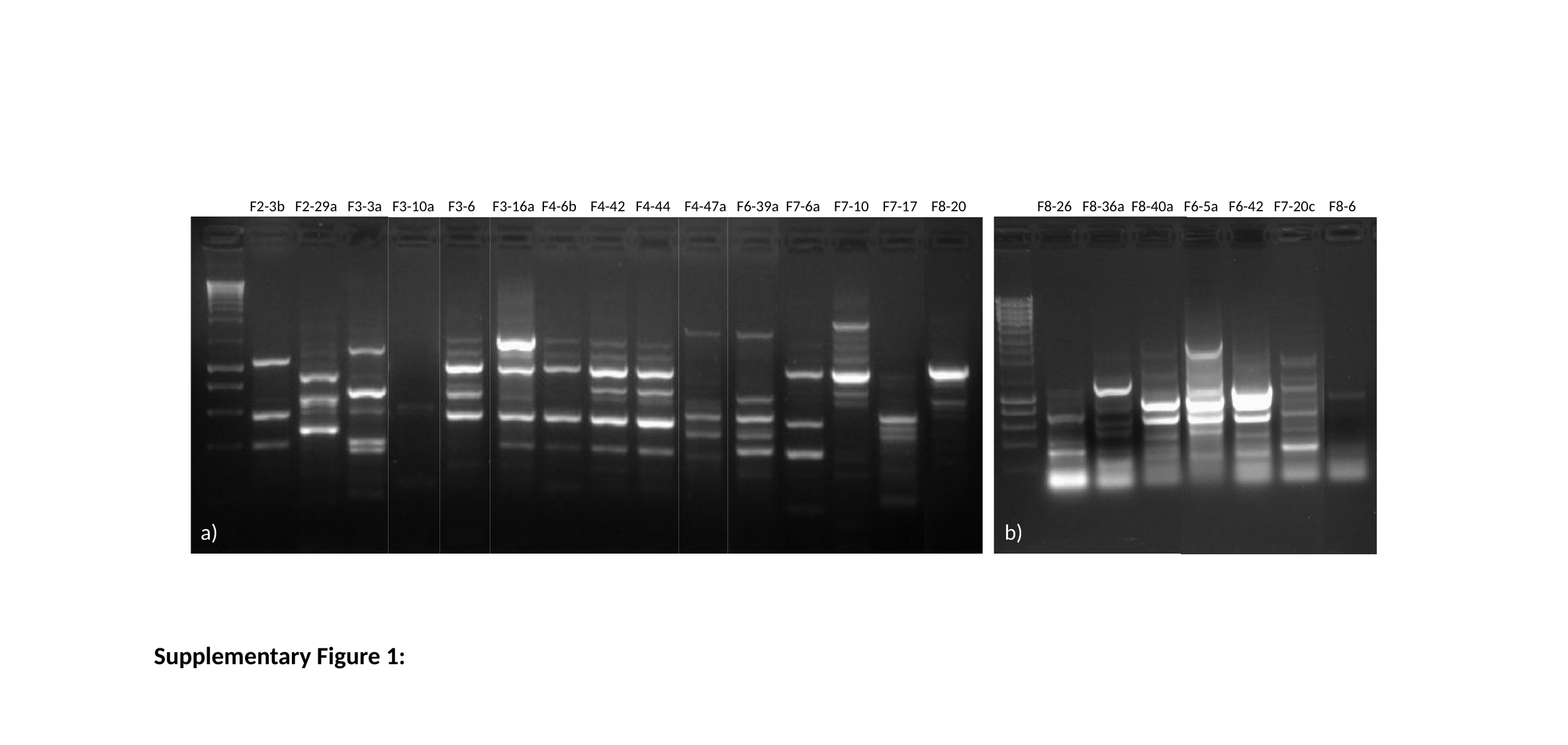

F2-3b F2-29a F3-3a F3-10a F3-6 F3-16a F4-6b F4-42 F4-44 F4-47a F6-39a F7-6a F7-10 F7-17 F8-20
F8-26 F8-36a F8-40a F6-5a F6-42 F7-20c F8-6
a)
b)
Supplementary Figure 1:

Supplement: Additional file 1: Figure S1 — ERIC-PCR profiling of: Pseudomonas aeruginosa strains f2-3b, faro2 29a, faro3 3a, faro3 6, faro3 10a, faro3 16a, faro4 6b, faro4 42, faro4 44, faro4 47a, faro6 39a, faro 7 6a and faro7 10, faro 7 17 and faro8 20, figure a) from left to right. On figure b) the strains P. aeruginosa faro8 26, faro8 36a, faro8 40a, faro6 5a, faro6 42, faro7 20c and faro8 6. Samples loaded on electrophoresis gel 1% agarose, 70 V, 60 min, stained with ethidium bromide. [file 1471-2180-14-118-S1.pptx]
